# The Putative RNA Methyltransferase Modulates T3SS Expression and Host NF-κB Activation via T6SS-Mediated Translocation in *Pseudomonas aeruginosa*

**DOI:** 10.3390/ijms27020818

**Published:** 2026-01-14

**Authors:** YuRim An, Yeji Lee, Yongxin Jin, Weihui Wu, Un-Hwan Ha

**Affiliations:** 1 Department of Biotechnology and Bioinformatics, Korea University, Sejong 30019, Republic of Korea; ayr7777@korea.ac.kr (Y.A.); yejee90@korea.ac.kr (Y.L.); 2 Interdisciplinary Graduate Program for Artificial Intelligence Smart Convergence Technology, Korea University, Sejong 30019, Republic of Korea; 3 State Key Laboratory of Medicinal Chemical Biology, Key Laboratory of Molecular Microbiology and Technology of the Ministry of Education, Department of Microbiology, Nankai University, Tianjin 300071, China; yxjin@nankai.edu.cn (Y.J.); wuweihui@nankai.edu.cn (W.W.)

**Keywords:** NF-κB, PA3840, *Pseudomonas aeruginosa*, T3SS, T6SS, twitching motility

## Abstract

RNA methyltransferases are key regulators of bacterial physiology, yet their specific roles in virulence remain poorly defined. In this study, we characterize PA3840, a putative RNA methyltransferase in *Pseudomonas aeruginosa* (*P. aeruginosa*). Deletion of PA3840 specifically impaired twitching motility without affecting bacterial growth, swimming, or swarming. Notably, PA3840 was found to suppress the expression of Type III Secretion System (T3SS) genes, thereby reducing cytotoxicity and host cell rounding. Consistent with these observations, PA3840 expression attenuated pro-inflammatory cytokine production in epithelial cells by inhibiting NF-κB activation. Mechanistic analysis revealed that PA3840 is translocated into host cells in a Type VI Secretion System (T6SS)-dependent manner. This translocation was reduced by *hcp1* deletion and nearly abolished by a double deletion of *pscF* and *hcp3*, suggesting the involvement of multiple T6SS components and potential interplay with T3SS machinery. However, direct transfection of PA3840 into host cells failed to suppress cytokine expression, indicating that its immunomodulatory function is mediated by a bacterium-intrinsic mechanism rather than direct intracellular action. Collectively, these findings identify PA3840 as a translocated effector that modulates twitching motility and dampens host inflammation by repressing T3SS and NF-κB signaling, revealing a novel layer of post-transcriptional virulence regulation in *P. aeruginosa*.

## 1. Introduction

*Pseudomonas aeruginosa* (*P. aeruginosa*) is a versatile opportunistic pathogen capable of causing a broad spectrum of diseases, most notably acute and chronic respiratory infections [1,2]. During the infection process, *P. aeruginosa* adheres to the respiratory epithelium and elicits a robust inflammatory response; if unresolved, this inflammation can lead to progressive lung damage and respiratory failure. These infections primarily affect immunocompromised individuals, including burn patients and those undergoing catheterization or mechanical ventilation, as well as individuals with cystic fibrosis (CF) [3,4]. Treatment is increasingly challenging due to the bacterium’s intrinsic resistance to multiple antibiotics [5], leading the World Health Organization to designate it a high-priority pathogen (WHO, 2024 [6]). Beyond its resistance profile, *P. aeruginosa* employs diverse virulence strategies to colonize and invade host tissues. These include a single polar flagellum that facilitates near-surface swimming and initial attachment, and retractile Type IV pili that mediate surface adhesion and twitching motility. Furthermore, the bacterium secretes an extensive array of virulence factors, which are either released into the extracellular milieu or delivered directly into host cells via specialized secretion systems [7,8].

Among these specialized delivery mechanisms, the Type III Secretion System (T3SS) is critical for the establishment of acute infections. Upon contact with host cells, the T3SS injects effector proteins that subvert host cellular processes to promote bacterial survival and dissemination [9,10]. This system is transcriptionally governed by the master regulator ExsA [11], which drives the expression and translocation of key effectors, including ExoS, ExoT, ExoY, ExoU, and Ndk [10,12]. In contrast, the Type VI Secretion System (T6SS) delivers a broader range of effectors into both eukaryotic and prokaryotic targets, contributing to host tissue damage and interbacterial competition [13,14]. *P. aeruginosa* encodes four distinct T6SS clusters—H1-, H2-, H3-, and H4-T6SS—which can act cooperatively to facilitate niche colonization [15]. The H1-T6SS is primarily associated with antibacterial activity and is often inversely regulated with the T3SS during the transition to chronic infection. Conversely, the H2- and H3-T6SS contribute more directly to host cell toxicity and overall virulence [16]. The recently identified H4-T6SS is phylogenetically distinct and does not share a recent common ancestor with the other three clusters [15].

The H1-T6SS is the most thoroughly characterized of the three systems and delivers well-defined antibacterial effectors, such as Tse1–Tse3 [13]. In contrast, the H2- and H3-T6SSs exhibit dual specificity, targeting both bacterial and eukaryotic cells; however, their precise effector repertoires and delivery mechanisms remain less understood. T6SS effectors are generally associated with structural components—such as Hcp, VgrG, or PAAR proteins—and are delivered either via direct protein–protein interactions or through the assistance of specialized chaperones and adaptor proteins [17,18]. For instance, the proper docking of the VgrG spike requires interaction with the Hcp tube [19]. While Hcp1 within the H1-T6SS is the most extensively studied Hcp homolog in *P. aeruginosa*, other isoforms likely support the secretion of distinct, system-specific effectors.

Beyond these secreted effectors, *P. aeruginosa* utilizes a diverse array of virulence factors that facilitate successful infection and pathogenesis. For instance, the water-soluble pigment pyocyanin targets the mitochondrial respiratory chain of neutrophils, generating reactive oxygen species that induce apoptosis and produce the pathogen’s characteristic green-colored pus [20]. Additionally, a broad suite of lytic enzymes—including LasA and LasB elastases, alkaline protease (AprA), LipC lipases, phospholipase C, and esterase A—modulates various virulence mechanisms [2,21]. Furthermore, cell surface components such as lipopolysaccharide and quorum-sensing systems enhance toxicity and facilitate colonization through complex inter-bacterial interactions [22,23].

In response to these *P. aeruginosa* virulence factors, the host elicits a robust inflammatory response essential for initiating immune defense. This process involves the activation of key regulatory pathways—primarily the inhibitor of κB kinase (IKK/NF-κB) and mitogen-activated protein kinase (MAPK) pathways—which control the expression of pro-inflammatory cytokines and chemokines, including IL-1β, IL-6, IL-8, and TNF-α [24]. As prototypical pro-inflammatory cytokines predominantly regulated by NF-κB, IL-1β and TNF-α activate neighboring cells and amplify inflammatory signaling, though they also contribute to sepsis and tissue damage [25]. Meanwhile, IL-6 is induced mainly via the p38 MAPK and NF-κB pathways to mediate systemic inflammation, regulate the acute-phase response, and support immune cell differentiation [26]. Finally, the neutrophil-attracting chemokine IL-8 (CXCL8) is strongly induced by integrated NF-κB and MAPK signaling [27]. This orchestration results in massive neutrophil recruitment and transmigration across vascular endothelial barriers, a hallmark of the acute purulent inflammation observed in *P. aeruginosa* pneumonia [28].

Although RNA methyltransferases are not traditionally classified as virulence factors, translational regulation is essential for bacterial pathogenicity. In *P. aeruginosa* strain PA14, the methyltransferase PrmC modulates global virulence pathways—including motility, T3SS activity, and anaerobic adaptation—by modifying translation termination factors [28]. In this study, we investigated the locus PA3840, which is annotated as a conserved hypothetical protein and a putative RNA methyltransferase (Pseudomonas Genome Database). PA3840 shares approximately 55.5% amino acid identity with the *Escherichia coli* (*E. coli*) ortholog RlmF. Based on this homology, PA3840 is predicted to participate in 23S ribosomal RNA (rRNA) modification, potentially fine-tuning ribosome biogenesis and translational efficiency under environmental stress or host immune pressure. Although PA3840 was previously identified as an in vivo–induced gene during infection in a murine burn model [29], its specific functional role in *P. aeruginosa* remains uncharacterized. Here, we evaluate its impact on bacterial growth, motility, virulence, and host immune evasion. Furthermore, we demonstrate that PA3840 is translocated into host cells via a T6SS-dependent mechanism, potentially involving cross-talk with the T3SS machinery. Our findings reveal a novel role for PA3840 in modulating host–pathogen interactions and the post-transcriptional regulation of virulence in *P. aeruginosa*.

## 2. Results

### 2.1. Differential Effects of PA3840 on Bacterial Growth and Motility

**Figure 1:**
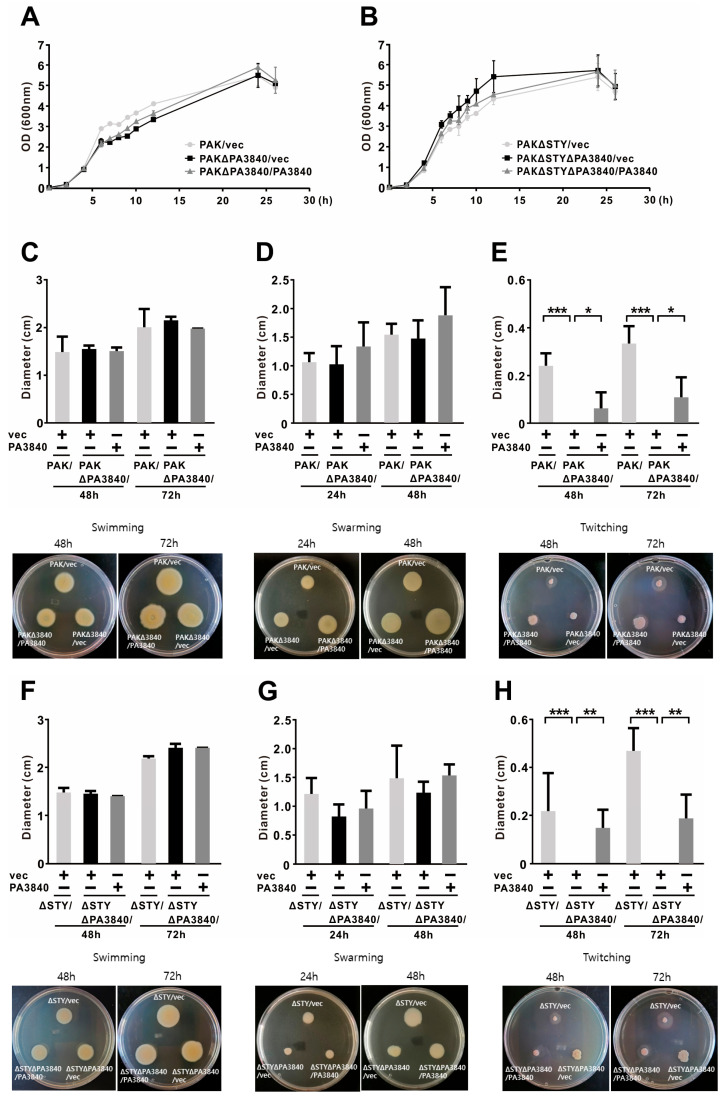
Differential effects of PA3840 on bacterial growth and motility. PA3840 deletion mutants in the background of PAK wt (PAKΔPA3840; (**A**,**C**–**E**) and PAKΔSTY (PAKΔSTYΔPA3840; (**B**,**F**–**H**)), carrying either an empty vector (vec) or a PA3840-flag expression plasmid (PA3840), were used. (**A**,**B**) Bacterial growth was measured by culturing the strains in LB with appropriate antibiotics over the indicated time points. (**C**–**H**) Motility assays were performed using the same strains to assess swimming (0.3% of LB over 48 and 72 h; (**C**,**F**)), swarming (0.5% of LB over 24 and 48 h; (**D**,**G**)), and twitching motility (1.5% of LB over 48 and 72 h; (**E**,**H**)). Data are presented as mean ± SD from three independent experiments. *, *p* < 0.05; **, *p* < 0.01; ***, *p* < 0.001.

*P. aeruginosa* pathogenesis relies on an integrated suite of strategies—including robust growth, surface adhesion, and the secretion of virulence factors—that facilitate adaptation to hostile host environments [30]. To investigate the role of PA3840 in bacterial physiology, we constructed a PA3840 deletion mutant (PAKΔPA3840) in the wild-type (WT) PAK background. Growth kinetics of PAK/vec, PAKΔPA3840/vec, and the complemented strain PAKΔPA3840/PA3840 were comparable (Figure 1A), indicating that PA3840 is not required for bacterial proliferation. Similar results were observed in a PAKΔSTY background using the PAKΔSTYΔPA3840 mutant (Figure 1B), confirming that PA3840 is dispensable for growth under these experimental conditions.

We next evaluated the contribution of PA3840 to bacterial motility. *P. aeruginosa* exhibits three distinct modes of movement—swimming, swarming, and twitching—each driven by unique mechanical systems and associated with specific stages of infection [31]. As shown in Figure 1C–H, the deletion of PA3840 had no discernible effect on swimming or swarming motility in either genetic background. In striking contrast, twitching motility was completely abolished in the PA3840 deletion strains. This phenotype was fully restored upon complementation, highlighting a specific and essential requirement for PA3840 in Type IV pili-mediated twitching.

### 2.2. PA3840 Suppresses T3SS Effector Gene Expression

**Figure 2:**
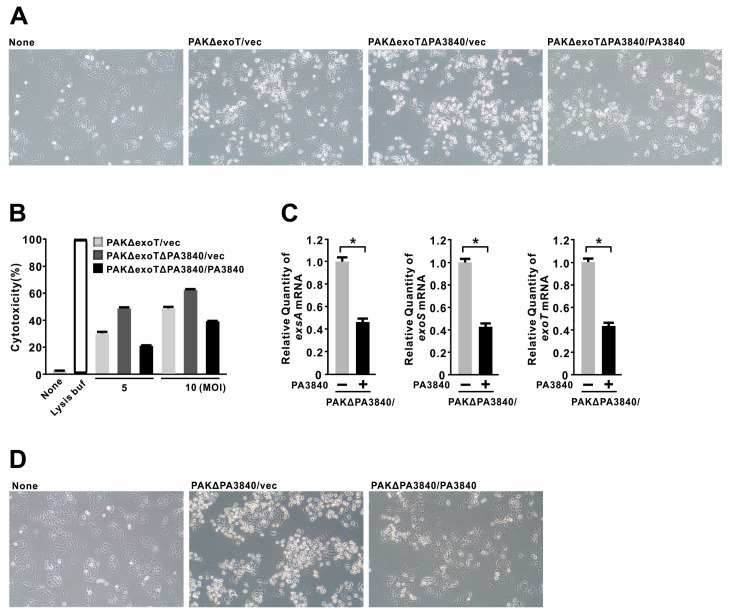
PA3840 suppresses T3SS effector gene expression. (**A**,**B**) A549 cells were infected with a PAKΔexoT strain carrying either vec or the PA3840 deletion mutant (PAKΔexoTΔPA3840) complemented with either vec or PA3840. Morphological changes were observed after 3 h of infection at MOI 5 (**A**; magnification, ×100), and LDH release was quantified to assess cytotoxicity after infection at MOI 5 or 10 for 7 h (**B**). (**C**) PAKΔPA3840 mutants carrying either vec (−) or PA3840 (+) were grown in LB with antibiotics for 3 h, and qRT-PCR was performed to assess the expression of T3SS-related genes. (**D**) A549 cells were infected with the same strains (MOI 5, 3 h), and morphological changes were monitored (magnification, ×100). Data in (**A**,**D**) represent three independent experiments. Data in (**B**,**C**) are presented as mean ± SD from three independent repeats. *, *p* < 0.05.

Given its significant impact on twitching motility, we next investigated whether PA3840 influences *P. aeruginosa* virulence. For these assays, we utilized a PAKΔexoT strain (which retains ExoS activity) to infect A549 cells, a widely used human alveolar epithelial cell line. As expected, infection led to characteristic host cell rounding (Figure 2A). Interestingly, the deletion of PA3840 (PAKΔexoTΔPA3840) further enhanced cell rounding, whereas complementation reversed this phenotype. Cytotoxicity, quantified by lactate dehydrogenase (LDH) release, was similarly reduced upon complementation of PA3840 (Figure 2B). Gene expression analysis further revealed that PA3840 complementation suppressed the mRNA levels of core T3SS-related genes (*exsA*, *exoS*, and *exoT*) by approximately 0.43–0.46-fold (Figure 2C). Consistent with these findings, PA3840 deletion in the wild-type background (PAKΔPA3840) also enhanced cell rounding in infected A549 cells (Figure 2D). Collectively, these data support a role for PA3840 as a negative regulator of T3SS effector expression and subsequent host cell damage.

### 2.3. PA3840 Attenuates Host Inflammatory Responses via NF-κB Inhibition

**Figure 3:**
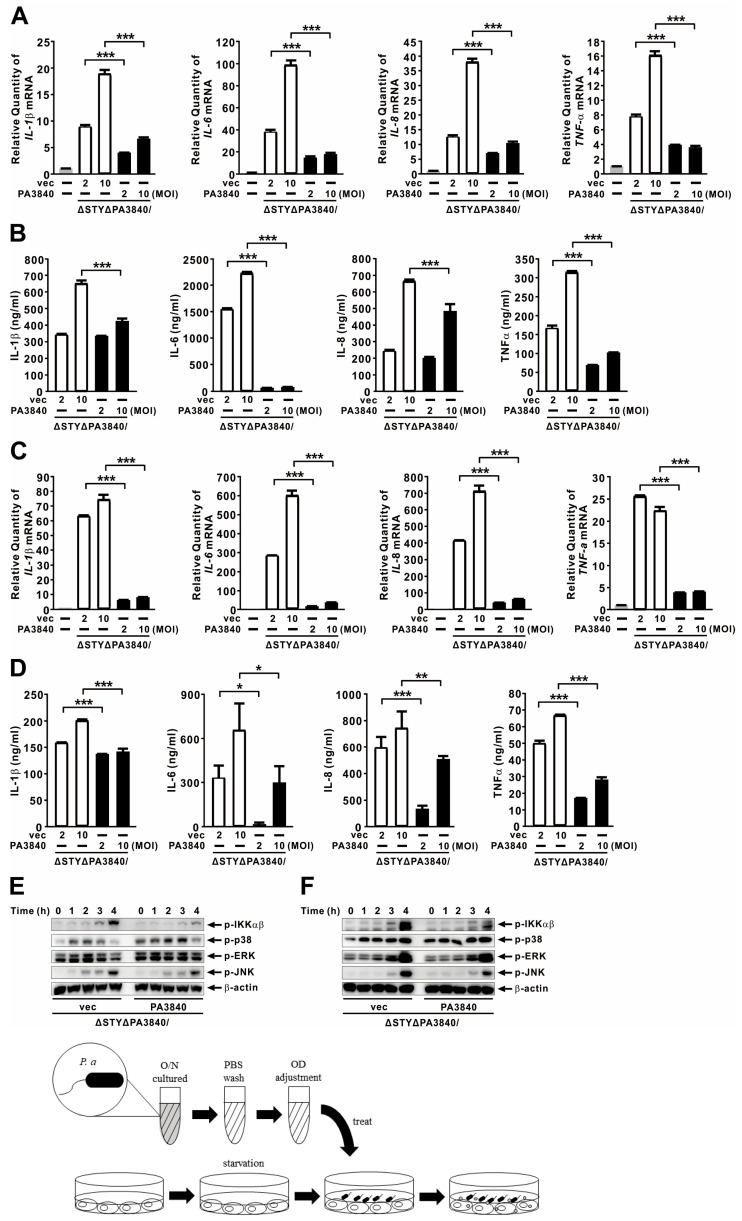
PA3840 attenuates host inflammatory responses via NF-κB inhibition. PAKΔSTYΔPA3840 mutants expressing either vec or PA3840 were used to infect cells. (**A**–**D**) A549 (**A**,**B**) and BEAS-2B (**C**,**D**) cells were infected at MOI 2 or 10 for 4 h (**A**,**C**) or 6 h (**B**,**D**). mRNA levels were quantified by qRT-PCR analysis (**A**,**C**), and protein levels were assessed by ELISA (**B**,**D**). A schematic diagram is shown above to clearly illustrate the experimental process. (**E**,**F**) Immunoblot analysis was performed after infection of A549 (**E**) and BEAS-2B (**F**) cells at MOI 10 for the indicated time points. Data in (**A**–**D**) are presented as mean ± SD from three independent repeats. Dara in (**E**,**F**) are representative of three independent experiments. *, *p* < 0.05; **, *p* < 0.01; ***, *p* < 0.001.

To assess the immunomodulatory effects of PA3840, we utilized the PAKΔSTYΔPA3840 mutant to minimize T3SS effector-induced cytotoxicity, which can otherwise confound host response analysis. In infected A549 cells, the presence of PA3840 significantly reduced the mRNA expression of IL-1β, IL-6, IL-8, and TNF-α (Figure 3A). At the protein level, IL-6 and TNF-α showed corresponding reductions; however, while the decreases in IL-1β and IL-8 protein levels reached statistical significance, they were not strictly proportional to their mRNA declines (Figure 3B). Similar inhibitory effects were observed in BEAS-2B cells, a non-tumorigenic human bronchial epithelial cell line (Figure 3C,D), suggesting that PA3840 suppresses cytokine production across both alveolar and bronchial epithelia. Pro-inflammatory cytokine production is frequently mediated by the NF-κB and MAPK signaling pathways [32]. Mechanistically, we found that PA3840 selectively inhibited the phosphorylation of IKKα/β—core components of the NF-κB activation complex—without affecting the activation of p38, ERK, or JNK MAPKs (Figure 3E,F). These data suggest that PA3840 exerts its anti-inflammatory effects primarily through the targeted inhibition of the NF-κB signaling pathway.

### 2.4. PA3840 Is Translocated into Host Cells via T6SS

**Figure 4:**
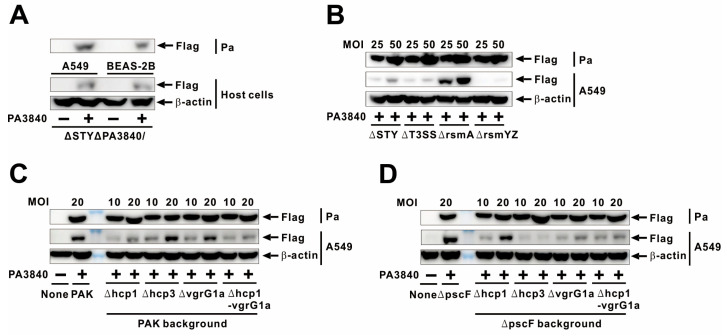
PA3840 is translocated into host cells via T6SS. (**A**) A549 and BEAS-2B cells were infected with PAKΔSTYΔPA3840 strains carrying either vec (−) or PA3840 (+) at MOI 10 for 4 h. (**B**) A549 cells were infected with mutants (ΔSTY, ΔT3SS, ΔrsmA, and ΔrsmYZ) carrying PA3840 (+) at MOI of 25 or 50 for 4 h. (**C**,**D**) A549 cells were infected with mutants (Δhcp1, Δhcp3, ΔvgrG1a, and Δhcp1ΔvgrG1a) in the PAK wt and PAKΔpscF background carrying PA3840 (+) at MOI 10 or 20 for 3 h. Protein levels were analyzed by immunoblotting. The immunoblot data are representative of three independent experiments.

Given the genomic proximity of PA3840 to exoS (PA3841) and their shared ExsA-dependent regulation [33], we hypothesized that PA3840 might function as a secreted effector. To test this, we infected A549 and BEAS-2B cells with PAKΔSTYΔPA3840 strains expressing Flag-tagged PA3840; immunoblot analysis of the host cytoplasmic fraction revealed clear translocation of the protein (Figure 4A). We then investigated the specific secretion machinery required for this translocation using mutants deficient in either the T3SS or T6SS. As shown in Figure 4B, PA3840 expression was consistent across all bacterial strains, and its translocation into host cells occurred in a dose-dependent manner. Notably, translocation persisted in a T3SS-null mutant (PAKΔT3SS). In contrast, translocation was markedly enhanced in the T6SS-active PAKΔrsmA strain but abolished in the T6SS-defective PAKΔrsmYZ mutant, strongly implying a T6SS-dependent delivery mechanism. To dissect the contribution of specific T6SS components, we evaluated mutants lacking Hcp1, Hcp3, or VgrG1a. While PA3840 translocation remained intact in the PAKΔhcp3 and PAKΔvgrG1a strains, it was significantly reduced in the PAKΔhcp1 mutant and further diminished in the PAKΔhcp1ΔvgrG1a double mutant (Figure 4C), identifying Hcp1 as a critical structural requirement for secretion. Given that PA3840 translocation was more severely reduced in the PAKΔT3SS strain than in the PAKΔSTY strain (Figure 4B), we further explored potential T3SS–T6SS interplay by introducing these mutations into a PAKΔpscF background, which lacks the T3SS needle apparatus. Interestingly, PA3840 translocation persisted in the PAKΔpscF background, confirming the T6SS as the primary delivery route. However, translocation was further attenuated in double mutants lacking Hcp3 or VgrG1a (Figure 4D) compared to the single mutants in the wild-type background. Notably, translocation was actually enhanced in the PAKΔpscFΔhcp1 mutant relative to PAKΔhcp1 alone, suggesting a possible compensatory regulation or physical cross-talk between T3SS and T6SS components. Collectively, these data indicate that PA3840 is primarily translocated via the T6SS, involving multiple structural components and potential coordination with the T3SS machinery.

### 2.5. PA3840 Does Not Directly Modulate Cytokine Expression in Host Cells

**Figure 5:**
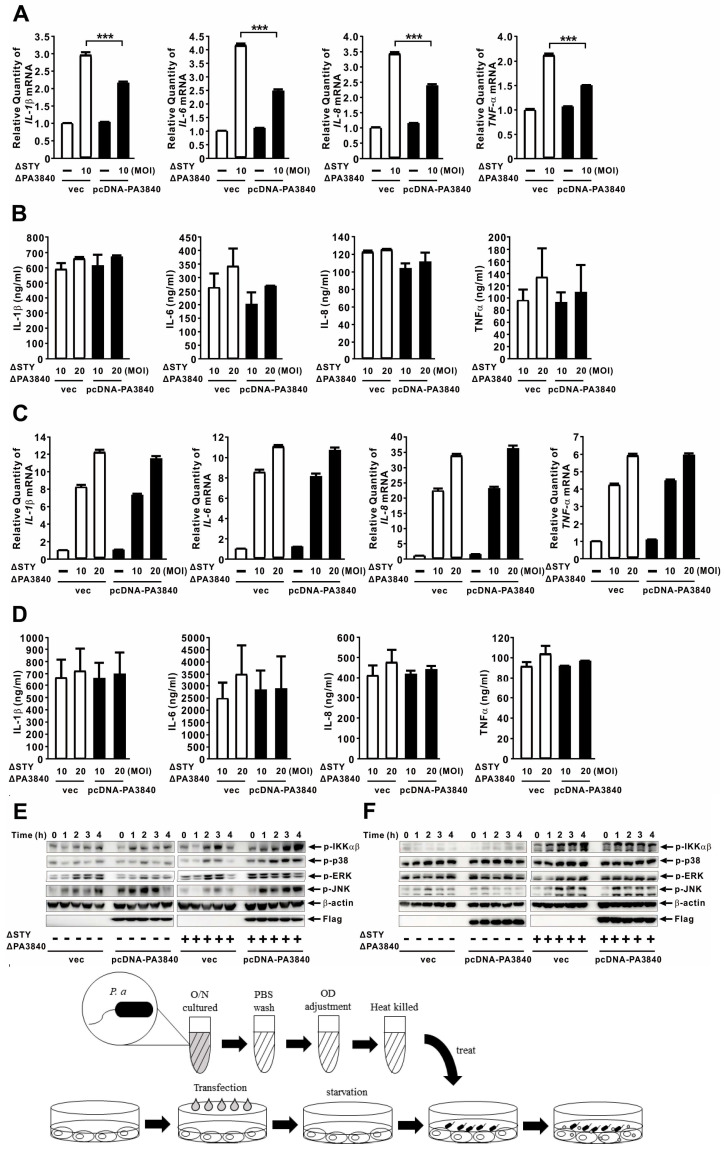
PA3840 does not directly modulate cytokine expression in host cells. Cells were transfected with either vec or pcDNA-PA3840 for 24h before infection. (**A**–**D**) A549 (**A**,**B**) and BEAS-2B (**C**,**D**) cells were infected with PAKΔSTYΔPA3840 at MOI 10 or 20 for 4 h (**A**,**C**) or 6 h (**B**,**D**). mRNA expression was analyzed by qRT-PCR (**A**,**C**), and protein levels were quantified by ELISA (**B**,**D**). A schematic diagram is shown above to clearly illustrate the experimental process. (**E**,**F**) A549 (**E**) and BEAS-2B (**F**) cells were infected at MOI 10 for the indicated times, and protein levels were assessed by immunoblotting. Data in (**A**–**D**) are presented as mean ± SD from three independent experiments. Dara in (**E**,**F**) are representative of three independent experiments. ***, *p* < 0.001.

To determine whether PA3840 directly modulates host responses as an intracellular effector, we transfected A549 and BEAS-2B cells with a pcDNA-PA3840 plasmid and subsequently stimulated them with PAKΔSTYΔPA3840 infection. In A549 cells, only a modest reduction in cytokine mRNA was observed (Figure 5A). However, cytokine protein levels remained unchanged and failed to exhibit a dose-dependent response (Figure 5B); similar results were observed in BEAS-2B cells (Figure 5C,D). To assess the activation of downstream signaling, we measured the phosphorylation of IKKα/β and MAPKs following pcDNA-PA3840 transfection. Despite efficient transfection (confirmed by Flag-tag detection), intracellular expression of PA3840 alone did not significantly alter phosphorylation patterns, with the exception of minor modulations in ERK and JNK. Upon subsequent infection, the phosphorylation of NF-κB and MAPKs was only mildly enhanced in A549 cells and remained unchanged in BEAS-2B cells (Figure 5E,F). These findings suggest that PA3840 does not directly modulate host cytokine production in a cell-autonomous manner once inside the host cytoplasm. Instead, its immunomodulatory effects appear to be mediated through bacterium-intrinsic processes or interactions occurring during the infection and translocation process itself.

## 3. Discussion

In this study, we characterized the functional role of PA3840 in *P. aeruginosa* physiology and host interaction. Our findings reveal that PA3840 is indispensable for Type IV pili-mediated twitching motility, yet it has no discernible effect on flagella-driven swimming or swarming—behaviors typically associated with acute infection [34]. Given that twitching is essential for epithelial cytotoxicity [35] and early-stage biofilm formation [36], PA3840 likely contributes to pathogenesis by regulating these processes. Notably, PA3840 is an in vivo–induced gene [29] associated with early-stage CF airway infections [37], further underscoring its clinical relevance. Additionally, our results demonstrate that while PA3840 acts as a negative regulator of the T3SS, its immunomodulatory effects appear to arise from bacterium-intrinsic regulatory shifts rather than direct intracellular interference with host signaling pathways.

Based on sequence homology, PA3840 is annotated as a putative RNA methyltransferase that regulates protein synthesis by modulating ribosome biogenesis and translational efficiency. Recent advances in bacterial epitranscriptomics suggest that RNA modifications serve as critical post-transcriptional checkpoints for various virulence factors [38,39]. In line with these findings, translational regulation in *P. aeruginosa* may facilitate the rapid and energy-efficient modulation of virulence factor production during infection. However, it is important to emphasize that direct biochemical or functional validation of methyltransferase activity was not performed in this study. Consequently, any proposed link between PA3840, ribosome biogenesis, and translational efficiency remains hypothetical. Nonetheless, PA3840 may modulate the translation of transcripts essential for Type IV pili assembly and T3SS regulation; however, whether this occurs via direct enzymatic RNA modification or an indirect regulatory mechanism warrants further investigation.

A key observation in our study is that PA3840 acts as a negative regulator of the T3SS; its presence correlates with the reduced expression of *exsA*, *exoS*, and *exoT*, likely at the post-transcriptional level. This aligns with previous studies identifying PA3840 as a protein that antagonizes the master regulator ExsA to limit T3SS gene expression [29]. Interestingly, we found that PA3840 is translocated into host cells via the T6SS. This translocation event may relieve *exsA* repression and subsequently promote T3SS gene expression, a mechanism analogous to that of ExsE. In the ExsE system, the secretion of the negative regulator frees the master regulator ExsA to activate T3SS transcription [40]. Collectively, our results support a model where PA3840 represses T3SS activity within the bacterium; the subsequent secretion of PA3840, facilitated by the T6SS, may then relieve this repression during the course of infection.

*P. aeruginosa* is recently proposed to encode four distinct T6SS clusters—H1-, H2-, H3-, and H4-T6SS—each characterized by specialized structural components and unique effector repertoires [13,15]. The Hcp and VgrG proteins serve as core structural components essential for the assembly of the contractile tail and the subsequent delivery of effectors. The RNA-binding protein RsmA acts as a post-transcriptional repressor of specific target transcripts [41]. Because RsmA represses the H1- and H3-T6SS clusters but does not exert the same control over H2-T6SS [41], the robust translocation of PA3840 observed in an rsmA mutant indicates a dependence on either the H1- or H3-T6SS. Furthermore, PA3840 translocation was significantly reduced in PAKΔhcp1 and PAKΔhcp1ΔvgrG1a mutants, suggesting a primary dependence on the H1-T6SS. Strikingly, translocation was nearly abolished in the PAKΔpscFΔhcp3 double mutant. Conversely, translocation was higher in the PAKΔpscFΔhcp1 double mutant than in the PAKΔhcp1 single mutant. Although PscF is a core component of the T3SS needle apparatus [42], its deletion appears to modulate T6SS activity, pointing toward a compensatory physiological shift rather than a direct physical interaction between the two machineries. While the precise mechanisms underlying this compensatory regulation remain to be elucidated, previous studies have highlighted the synergistic roles of the T3SS and T6SS in promoting bacterial fitness and virulence [43].

The reciprocal modulation of the T3SS and T6SS suggests a sophisticated coordination of bacterial resources. Such trade-offs are increasingly recognized as a hallmark of *P. aeruginosa* adaptation; indeed, recent single-cell analyses confirm that the T3SS and H1-T6SS often exhibit antagonistic expression patterns, potentially mediated by fluctuations in secondary messengers such as c-di-GMP [44]. Furthermore, the down-regulation of the T3SS is often a consequence of metabolic specialization, where the bacterium prioritizes energy-efficient persistence over acute cytotoxicity [45]. Within this context, PA3840 may function as a regulatory rheostat that assists the cell in navigating these energetic demands during the transition to a more persistent infection mode [14]. Moreover, inactivation of the H3-T6SS component ClpV3 significantly reduces the expression of T3SS genes, such as *exoY* and *exsD*, establishing a clear regulatory link between these systems [46]. Additionally, while most T6SS effectors are cluster-specific, certain proteins—such as Tse6 and PldA/B—exhibit ‘promiscuity’ and can be secreted via multiple T6SS clusters [15]. Collectively, these observations suggest that PA3840 may utilize a flexible, cross-system secretion mechanism involving components from both the T3SS and T6SS. Such functional redundancy likely enhances the adaptability and persistence of *P. aeruginosa* across diverse host environments.

A central challenge in interpreting our data lies in the biological relevance of PA3840 translocation. While PA3840 is delivered to the host cytoplasm, intracellular expression of the protein via transfection failed to reproduce the suppression of NF-κB signaling or cytokine production observed during live infection. This discrepancy suggests that PA3840 does not function as a classical ‘intracellular effector’ that directly targets host signaling proteins. Instead, its immunomodulatory effects are likely an indirect consequence of its regulatory role within the bacterium. Among the diverse virulence mechanisms of *P. aeruginosa*, the T3SS serves as a primary determinant of acute infection. This system injects cytotoxins, such as ExoS and ExoT, directly into host cells, where they disrupt cytoskeletal integrity and facilitate immune evasion [47,48]. By suppressing the expression of these T3SS effectors, PA3840 reduces the overall cytotoxic burden on the host cell. The observed inhibition of the IKKα/β complex and the subsequent reduction in IL-6, IL-8, and TNF-α should, therefore, be interpreted as a reflection of an attenuated bacterial virulence state rather than a direct intracellular action of PA3840 on the host NF-κB pathway. This immunomodulatory effect potentially fosters a more permissive environment for bacterial persistence, likely aiding in immune evasion during the course of infection.

Notably, we observed a discrepancy between the mRNA and protein expression levels of IL-1β and IL-8. This variance is likely attributable to complex post-transcriptional and post-translational regulatory mechanisms, including differences in mRNA and protein stability, translational control, and the kinetics of cumulative cytokine secretion. Such temporal uncoupling between transcriptional induction and protein abundance is a well-documented phenomenon in pro-inflammatory cytokine signaling. Interestingly, we also noted subtle differences in cytokine expression profiles and the activation of signaling mediators between A549 and BEAS-2B cells, which represent alveolar and bronchial epithelial origins, respectively. These differences likely reflect the distinct transcriptional landscapes of these cell types, including the differential expression of key transcription factors. Supporting this observation, A549 cells are known to be highly susceptible to respiratory syncytial virus (RSV) infection, whereas BEAS-2B cells exhibit significantly greater resistance [49].

## 4. Materials and Methods

### 4.1. Bacterial Strains and Culture Conditions

The bacterial strains used in this study are listed in Table 1. All strains were cultured in LB broth (1% NaCl, 1% tryptone, and 0.5% yeast extract, *w*/*v*) or on LB agar plates at 37 °C. Plasmids were maintained by adding ampicillin (100 μg/mL for *E. coli*) or carbenicillin (150 μg/mL for *P. aeruginosa*). For infection experiments, bacteria were cultured overnight in LB containing the appropriate antibiotics at 37 °C. Cells were harvested by centrifugation at 12,000× *g*, washed with 1 mL PBS (phosphate-buffered saline), and centrifuged again at 12,000× *g* for 1 min. Pellets were resuspended in 1 mL PBS, and optical density at 600 nm (OD_600_) was measured after a 1:10 dilution in PBS. Bacteria were mixed with calculated OD or MOI based on OD_600_.

Chromosomal gene deletions were generated as previously described [50]. In brief, to delete *PA3840*, *exoT*, *hcp1*, *hcp3* and *vgrG1a*, upstream and downstream flanking regions were amplified by PCR using primers listed in Table 2. The resulting fragments were cloned into the suicide vector pEX18Tc and introduced into *P. aeruginosa* via homologous recombination. Double-cross mutants were selected following standard procedures. For mutant construction, tetracycline (10 μg/mL for *E. coli* S17) and tetracycline (50 μg/mL) with kanamycin (25 μg/mL) for *P. aeruginosa* were used for selection.

**Table 1:** Bacterial strains and plasmids.

Strains or Plasmids	Description	Source
*P. aeruginosa*		
PAK	*P. aeruginosa*, wild type	David Bradley
PAKΔPA3840	PAK derivative with chromosomal deletion of *PA3840*	This study
PAKΔrsmA	PAK derivative with chromosomal deletion of *rsmA*	[51]
PAKΔrsmYZ	PAK derivative with chromosomal deletion of *rmsY* and *rsmZ*	[51]
PAKΔSTY	PAK derivative with chromosomal deletion of *exoS*, *exoT*, and *exoY*	[52]
PAKΔSTYΔPA3840	PAKΔSTY derivative with chromosomal deletion of *PA3840*	This study
PAKΔexoT	PAK derivative with chromosomal deletion of *exoT*	[12]
PAKΔexoTΔPA3840	PAKΔexoT derivative with chromosomal deletion of *PA3840*	This study
PAKΔT3SS	PAK derivative with deletion of the whole T3SS region	Shouguang Jin
PAKΔpscF	PAK derivative with chromosomal deletion of *pscF*	[12]
PAKΔpscFΔhcp1	PAKΔpscF derivative with chromosomal deletion of *hcp1*	This study
PAKΔpscFΔhcp3	PAKΔpscF derivative with chromosomal deletion of *hcp3*	This study
PAKΔpscFΔvgrG1a	PAKΔpscF derivative with chromosomal deletion of *vgrG1a*	This study
PAKΔpscFΔhcp1ΔvgrG1a	PAKΔpscF derivative with chromosomal deletion of *hcp1* and *vgrG1a*	This study
PAKΔhcp1	PAK derivative with chromosomal deletion of *hcp1*	[53]
PAKΔhcp3	PAK derivative with chromosomal deletion of *hcp3*	This study
PAKΔvgrG1a	PAK derivative with chromosomal deletion of *vgrG1a*	[53]
PAKΔhcp1ΔvgrG1a	PAK derivative with chromosomal deletion of *hcp1* and *vgrG1a*	This study
Plasmids		
pEX18Tc	Broad-host-range gene replacement vector; Tc^r^	[50]
pUCP20	*Escherichia-Pseudomonas* shuttle vector; Ap^r^	[54]
PA3840	pUCP20 carrying 1.0 kb PA3840-flag; Ap^r^	This study
pcDNA3.1(+)	Eukaryotic expression vector containing CMV promoter; Ap^r^	Invitrogen
pcDNA-PA3840	pcDNA3.1(+) carrying 1.0 kb PA3840-flag; Ap^r^	This study

**Table 2:** Primers used to construct plasmids.

Primers	Sequence (5′-3′)
PA3840-flag F	GGAATTCACGGAAACCGAGAACATG
PA3840-flag R	AACTGCAGTCACTTGTCGTCATCGTCCTTGTAGTCTTCGCCCAGTGGTTCCAGGAGGGC
PA3840 dmt-A-F	GGAATTCCATC
PA3840 dmt-A-R	GCTCTAGAGAAGTCGATGCTCTGCCTG
PA3840 dmt-B-F	GCTCTAGACAGGGACAGAAGCAGAGCC
PA3840 dmt-B-R	CCCAAGCTTCAGAGTCCGTCTTTCG
hcp1 dmt-A-F	GGAATTCCGACGCGATCAAGTC
hcp1 dmt-A-R	GCTCTAGATTCCTCGGCATGAGTC
hcp1 dmt-B-F	GCTCTAGAGTCGACTACCAGCC
hcp1 dmt-B-R	CCCAAGCTTGTTGTGGTCCATCTC
hcp3 dmt-A-F	CGGGATCCCTCGACGAGATCATCG
hcp3 dmt-A-R	GCTCTAGAGTTTTCGTAGCCGACG
hcp3 dmt-B-F	GCTCTAGACGCAGAAGGACGACGGC
hcp3 dmt-B-R	CCCAAGCTTCACGCTGTTCTCGCG
vgrG1a dmt-A-F	CGAATTCGGTGAGCCTGCTGGATACC
vgrG1a dmt-A-R	CGGGATCCTAGGCGAACAACCGTC
vgrG1a dmt-B-F	CGGGATCCTTCAACCTCTACGCC
vgrG1a dmt-B-R	CCAAGCTTCTGAACCATCCGCTGTG

### 4.2. Plasmids and Transfection

The primers used for plasmid construction are listed in Table 2. The coding regions of PA3840 were amplified by PCR from *P. aeruginosa* genomic DNA. PCR was performed with denaturation at 95 °C, primer-specific annealing temperature, and elongation at 72 °C. The reverse primer for PA3840 included a C-terminal FLAG tag (underlined), which does not affect the protein’s structure or function. PCR products were cloned into the vectors listed in Table 1, and expression plasmids were transformed into *P. aeruginosa* strains. For eukaryotic expression, the PA3840-flag insert was cloned into the pcDNA3.1(+) vector (Invitrogen, Carlsbad, CA, USA). Plasmids were prepared using the EndoFree Plasmid Maxi kit (Qiagen, Valencia, CA, USA) per the manufacturer’s instructions. Transfection into mammalian cells was performed using 1.0 µg plasmid DNA and Lipofectamine™ 3000 (Invitrogen), followed by 24 h incubation in complete medium (10% FBS) at 37 °C.

### 4.3. Mammalian Cell Culture

A549 (human alveolar epithelial cells) were cultured in Roswell Park Memorial Institute 1640 (RPMI 1640; HyClone, Rockford, IL, USA), and BEAS-2B (human bronchial epithelial cells) in Dulbecco’s Modified Eagle’s Medium (DMEM; Corning, Glendale, AZ, USA), both supplemented with 10% heat-inactivated fetal bovine serum (FBS; HyClone), 100 units/mL penicillin, and 0.1 mg/mL streptomycin. Cells were maintained at 37 °C in a humidified 5% CO_2_ incubator.

### 4.4. Growth Assay

*P. aeruginosa* strains were grown overnight in LB with antibiotics at 37 °C. Cultures were diluted 1:10 in fresh LB to normalize starting OD_600_, then subcultured and monitored at indicated time points using a BioPhotometer D30 (Eppendorf, Hamburg, Germany). When OD_600_ exceeded 1.0, cultures were further diluted 1:10 for accurate measurement.

### 4.5. Motility Assay

Overnight cultures were normalized to OD_600_ = 1.0 before use. For the swimming motility assay, 5 µL of each strain was point-inoculated onto 0.3% LB agar plates containing 150 μg/mL carbenicillin and incubated at 30 °C [55]. For the swarming motility assay, 2 µL of culture was point-inoculated onto 0.5% LB agar plates with 150 μg/mL carbenicillin and incubated at 37 °C [56]. For the twitching motility assay, colonies were stab-inoculated into 1.5% LB agar plates with 150 μg/mL carbenicillin and incubated at 37 °C [57]. Motility diameters were measured at specific time points.

### 4.6. Cell Viability and Morphology

A549 and BEAS-2B cells were seeded at 4 × 10^5^ cells/well in 6-well plates and incubated at 37 °C with 5% CO_2_. Prior to infection, cells were serum- and antibiotic-starved for 1 h. Bacteria were added at the indicated MOI. Cell viability was assessed by LDH release using the CytoTox 96 Non-Radioactive Cytotoxicity assay (Promega, Madison, WI, USA). Culture supernatants were centrifuged at 12,000× *g* for 3 min, and 50 µL of the supernatant was mixed with 50 µL of reagent and incubated in the dark for 30 min. Absorbance was measured at 490 nm. Morphological changes were observed using a ×100 optical microscope (Zeiss, Jena, Germany).

### 4.7. Real-Time Quantitative PCR (qRT-PCR)

Primer sequences for qRT-PCR are listed in Table 3. Primer specificity was confirmed by melt curve analysis. Total RNA was isolated using TRIzol^®^ Reagent (Invitrogen) per the manufacturer’s instructions. RNA purity (A260/A280 ratio between 1.8 and 2.0) was assessed using a NanoDrop spectrophotometer (Thermo Fisher Scientific, Waltham, MA, USA). cDNA was synthesized using ReverTra Ace qPCR RT Master Mix with genomic DNA (gDNA) Remover (Toyobo, Osaka, Japan), which has DNase I that removes DNA before the transcription step, for bacteria or ReverTra Ace RT kit (Toyobo) for mammalian cells. qRT-PCR was performed using SYBR Green PCR Master Mix (KAPA Biosystems, Woburn, MA, USA) on a CFX96 Real-Time PCR System (Bio-Rad, Hercules, CA, USA). The cycling conditions were: 50 °C for 2 min, 95 °C for 10 min; followed by 40 cycles of 95 °C for 15 s and 60 °C for 1 min. Relative mRNA expression was calculated using the comparative CT method and normalized to bacterial 16S rRNA or human GAPDH.

### 4.8. Immunoblot Analysis

Mammalian cells were harvested with trypsin, washed with PBS, and lysed in 40 µL of lysis buffer (20 mM Tris-HCl, pH 7.4, 50 mM NaCl, 50 mM sodium pyrophosphate, 30 mM NaF, 5 µM ZnCl_2_, 2 mM iodoacetic acid, 1% Triton X-100, 1 mM PMSF, 0.1 mM sodium orthovanadate). Lysates were incubated on ice for 15 min and centrifuged at 14,000× *g* for 20 min at 4 °C. The resulting supernatant containing the total cytosolic protein was collected, and 5× sample buffer (250 mM Tris-HCl, pH 6.8, 0.1% bromophenol blue, 5% β-mercaptoethanol, 5% SDS, 20% Glycerol) was added. For the preparation of bacterial lysates, overnight-cultured bacterial cells were harvested by centrifugation at 12,000× *g* for 1 min. Bacterial lysates were prepared by resuspending pellets in 100 µL of 5× sample buffer. All protein samples were boiled for 10 min and resolved by SDS-PAGE. Protein concentrations were measured using the bicinchoninic acid (BCA) assay (Thermo Fisher Scientific, Waltham, MA, USA). Proteins were transferred to PVDF membranes (0.45 µm) and blocked with 5% non-fat milk in Tris-buffered saline (10 mM Tris-HCl, 150 mM NaCl) for 1 h. Membranes were incubated overnight at 4 °C with primary antibodies against FLAG, β-actin, p-IKKα/β, p-ERK, or p-p38 (Cell Signaling Technology, Danvers, MA, USA), followed by horseradish peroxidase (HRP)-conjugated secondary antibodies (Cell Signaling Technology). Detection was performed using SuperSignal™ West Pico PLUS/Femto Maximum Chemiluminescent Substrate (Thermo Fisher Scientific) and visualized using an Amersham ImageQuant 800 system (Cytiva, Marlborough, MA, USA).

### 4.9. Enzyme-Linked Immunosorbent Assay (ELISA)

A549 and BEAS-2B cells were seeded in 6-well plates and incubated at 37 °C in a humidified 5% CO_2_ incubator. Cells were serum-starved for 1 h at 37 °C before infection. After treatment with *P. aeruginosa*, supernatants were collected and centrifuged at 12,000× *g* for 5 min to remove impurities such as bacteria. Cytokine levels (IL-1β, IL-6, IL-8, TNF-α) were measured using human ELISA kits (R&D Systems, Minneapolis, MN, USA) according to the manufacturer’s instructions.

### 4.10. Statistics

All experiments were performed in triplicate. Data are presented as mean ± standard deviation (SD). Statistical significance was assessed using Student’s *t*-test or one-way ANOVA with Tukey’s post hoc test. A *p*-value < 0.05 was considered statistically significant.

## 5. Conclusions

**Figure 6:**
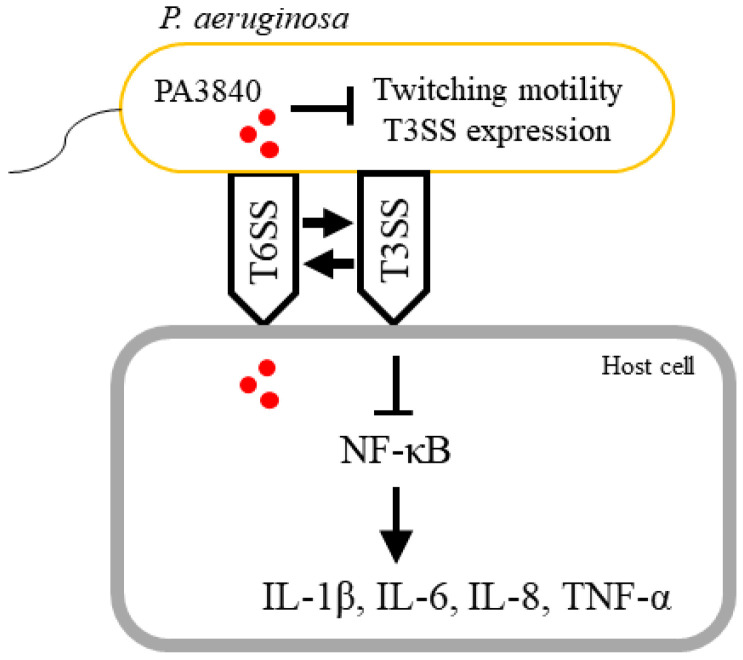
A schematic illustration depicting the proposed mechanism underlying PA3840-mediated modulation of motility and virulence in *P. aeruginosa*.

This study identifies PA3840 as a pivotal regulator of the *P. aeruginosa* virulence repertoire, specifically governing the transition between different modes of motility and immune modulation (Figure 6). Our data demonstrate that PA3840 is essential for Type IV pili-mediated twitching motility, yet remains dispensable for flagellar movement and general bacterial growth. Furthermore, we established that PA3840 functions as a negative regulator of the T3SS, as evidenced by the reduced expression upon PA3840 complementation. While we confirmed that PA3840 is translocated into host cells via the H1-T6SS in an Hcp1-dependent manner, its role as a direct intracellular effector remains unproven. The suppression of host NF-κB signaling and pro-inflammatory cytokine production observed during infection was not reproducible through direct intracellular expression of PA3840. This suggests that the immunomodulatory effects of PA3840 are likely an indirect consequence of its intrinsic bacterial regulatory activity—specifically its repression of T3SS-mediated cytotoxicity—rather than a direct manipulation of host signaling pathways. Although PA3840 is annotated as a putative RNA methyltransferase, its specific biochemical activity and the exact molecular mechanisms underlying the observed T3SS–T6SS cross-talk remain to be elucidated. Future studies should focus on the biochemical characterization of PA3840 to determine if its regulatory effects are mediated through direct RNA modification. Ultimately, our findings highlight PA3840 as a key internal checkpoint that coordinates bacterial fitness and virulence during host interaction, providing new insights into the complex regulatory networks of *P. aeruginosa*.

## Figures and Tables

**Table 3:** qRT-PCR Primers used in this study.

Primers	Sequence (5′-3′)
exoS F	CTCTACACCGGCATTCACTAC
exoS R	CATACCTTGGTCGATCAGCTT
exoT F	CTTCGAGGCGGTGAAAGAG
exoT R	GCCGAACAGGGTGGTTATC
exsA F	AAGGAGCCAAATCTCTTG
exsA R	CTTGTTTACCCTGTATTCG
*P. aeruginosa* 16S rRNA F	CAAAACTACTGAGCTAGAGTACG
*P. aeruginosa* 16S rRNA R	GCCACTGGTGTTCCTTCCTA
human IL-1β F	AAACAGATGAAGTGCTCCTTCCAG
human IL-1β R	TGGAGAACACCACTTGTTGCTCCA
human IL-6 F	AACCTGAACCTTCCAAAGATGG
human IL-6 R	TCTGGCTTGTTCCTCACTACT
human IL-8 F	CTTGGCAGCCTTCCTGATTT
human IL-8 R	GGGTGGAAAGGTTTGGAG
human TNF-α F	CAGAGGGAAGAGTTCCCCAG
human TNF-α R	CCTTGGTCTGGTAGGAGACG
human GAPDH F	CCCTCCAAAATCAAGTGG
human GAPDH R	CCATCCACAGTCTTCTGG

## Data Availability

The original contributions presented in this study are included in the article. Further inquiries can be directed to the corresponding author.
